# Gallionellaceae pangenomic analysis reveals insight into phylogeny, metabolic flexibility, and iron oxidation mechanisms

**DOI:** 10.1128/msystems.00038-23

**Published:** 2023-10-26

**Authors:** Rene L. Hoover, Jessica L. Keffer, Shawn W. Polson, Clara S. Chan

**Affiliations:** 1 Microbiology Graduate Program, University of Delaware, Newark, Delaware, USA; 2 Department of Earth Sciences, University of Delaware, Newark, Delaware, USA; 3 Department of Computer and Information Sciences, University of Delaware, Newark, Delaware, USA; 4 Center for Bioinformatics and Computational Biology, University of Delaware, Newark, Delaware, USA; 5 School of Marine Science and Policy, University of Delaware, Newark, Delaware, USA; University of Wisconsin-Milwaukee, Milwaukee, Wisconsin, USA

**Keywords:** iron oxidation, iron-oxidizing bacteria, pangenome, extracellular electron transfer, multiheme cytochrome

## Abstract

The iron-oxidizing Gallionellaceae drive a wide variety of biogeochemical cycles through their metabolisms and biominerals. To better understand the environmental impacts of Gallionellaceae, we need to improve our knowledge of their diversity and metabolisms, especially any novel iron oxidation mechanisms. Here, we used a pangenomic analysis of 103 genomes to resolve Gallionellaceae phylogeny and explore their genomic potential. Using a concatenated ribosomal protein tree and key gene patterns, we determined Gallionellaceae has four genera, divided into two groups: iron-oxidizing bacteria (FeOB) *Gallionella*, *Sideroxydans*, and *Ferriphaselus* with iron oxidation genes (*cyc2*, *mtoA*) and nitrite-oxidizing bacteria (NOB) *Candidatus* Nitrotoga with the nitrite oxidase gene *nxr*. The FeOB and NOB have similar electron transport chains, including genes for reverse electron transport and carbon fixation. Auxiliary energy metabolisms, including S oxidation, denitrification, and organotrophy, were scattered throughout the FeOB. Within FeOB, we found genes that may represent adaptations for iron oxidation, including a variety of extracellular electron uptake mechanisms. FeOB genomes encoded more predicted *c*-type cytochromes than NOB genomes, notably more multiheme *c*-type cytochromes (MHCs) with >10 CXXCH motifs. These include homologs of several predicted outer membrane porin-MHC complexes, including MtoAB and Uet. MHCs efficiently conduct electrons across longer distances and function across a wide range of redox potentials that overlap with mineral redox potentials, which can expand the range of usable iron substrates. Overall, the results of pangenome analyses suggest that the Gallionellaceae genera *Gallionella*, *Sideroxydans*, and *Ferriphaselus* have acquired a range of adaptations to succeed in various environments but are primarily iron oxidizers.

**IMPORTANCE:**

Neutrophilic iron-oxidizing bacteria (FeOB) produce copious iron (oxyhydr)oxides that can profoundly influence biogeochemical cycles, notably the fate of carbon and many metals. To fully understand environmental microbial iron oxidation, we need a thorough accounting of iron oxidation mechanisms. In this study, we show the Gallionellaceae FeOB genomes encode both characterized iron oxidases as well as uncharacterized multiheme cytochromes (MHCs). MHCs are predicted to transfer electrons from extracellular substrates and likely confer metabolic capabilities that help Gallionellaceae occupy a range of different iron- and mineral-rich niches. Gallionellaceae appear to specialize in iron oxidation, so it would be advantageous for them to have multiple mechanisms to oxidize various forms of iron, given the many iron minerals on Earth, as well as the physiological and kinetic challenges faced by FeOB. The multiple iron/mineral oxidation mechanisms may help drive the widespread ecological success of Gallionellaceae.

## INTRODUCTION

*Gallionella* are one of the oldest known and most well-studied iron-oxidizing bacteria (FeOB), yet we are still learning how they oxidize iron and adapt to iron-rich niches. *Gallionella* is the type genus of the family Gallionellaceae, which also includes *Sideroxydans*, *Ferriphaselus*, and *Ferrigenium*. These Gallionellaceae FeOB are found in a wide range of environments, including freshwater creeks, sediment, root rhizospheres, peat, permafrost, deep subsurface aquifers, and municipal waterworks (1–18). FeOB potentially drive the fate of many metals and nutrients via both metabolic reactions and forming iron oxyhydroxides that adsorb and react with many solutes (19). To better understand the biogeochemical effects of Gallionellaceae, we need to improve our knowledge of their phylogeny and metabolic mechanisms, especially for iron oxidation. Recently, the rapid increase in metagenomes from iron-rich environments has significantly expanded the number of available Gallionellaceae genomes, which makes it possible to investigate diversity and mechanisms using genomic analyses of both cultured and uncultured Gallionellaceae.

The Gallionellaceae are named after *Gallionella ferruginea*, first described by Ehrenberg in 1838 (20), and recognizable by its distinctive, twisted iron oxyhydroxide stalk (21). While the type strain, *G. ferruginea* Johan (22), no longer exists, there are seven iron-oxidizing Gallionellaceae isolates and several stable enrichment cultures (7, 11, 23–26). Some isolates, such as *Ferriphaselus* spp., appear to be obligate iron oxidizers, while others also grow on non-iron substrates. In addition to iron, *Sideroxydans lithotrophicus* ES-1 grows by thiosulfate oxidation (24, 27), while *Sideroxydans* sp. CL21 shows mixotrophic growth with either lactate or hydrogen (28). Some *Ferrigenium* are members of the stable autotrophic, nitrate-reducing, iron-oxidizing enrichment cultures Straub, Bremen Pond, and Altingen (29–32). It is unknown how common it is for Gallionellaceae to use electron donors/acceptors besides Fe(II)/O_2_, though these alternate metabolisms may help their success across different environments and fluctuating conditions typical of many oxic-anoxic interfaces. Even so, since these seven Gallionellaceae isolates are all neutrophilic aerobic chemolithoautotrophic iron oxidizers, this could be the dominant metabolic niche of Gallionellaceae.

In Gallionellaceae and other neutrophilic chemolithotrophic FeOB, there are two potential iron oxidases: Cyc2, a fused monoheme cytochrome-porin and MtoAB, a porin-decaheme cytochrome complex (33–35). Porin-cytochrome complexes conduct electrons across the outer membrane, allowing cells to oxidize Fe(II) outside the cell to avoid internal Fe(III) mineralization (36, 37). The *mtoA* (metal oxidation) gene was first identified and characterized in FeOB *S. lithotrophicus* ES-1 (33). It is a homolog of the well-studied *pioA* (phototrophic iron oxidation) iron oxidase gene for which the function was verified through genetic knockout in the photoferrotroph *Rhodopseudomonas palustris* TIE-1 (38). The *mtoA* gene is also a homolog of *mtrA* (metal reduction), which encodes the MtrA iron reductase in iron-reducing bacteria (FeRB) *Shewanella* (39). Though known for electron export, MtrA can also conduct electrons into the cell (40). The *cyc2* gene is more common than *mtoAB* and is found in nearly all well-characterized neutrophilic FeOB like the Gallionellaceae (41–43) and Zetaproteobacteria (43), making it a suitable genetic marker for many FeOB. Cyc2 has been demonstrated to oxidize aqueous Fe^2+^ (34). In *S. lithotrophicus* ES-1 cultures grown on aqueous Fe^2+^, *cyc2*/Cyc2 is highly expressed, whereas *mtoA* expression is low, and the Mto proteins are not detected, suggesting Cyc2 plays a larger role in aqueous iron oxidation compared to MtoA (27, 44). In contrast, Mto gene/protein expression is upregulated in ES-1 cultures grown on Fe(II) smectite clay, suggesting MtoAB plays a role in the oxidation of solid iron minerals (44). However, Cyc2 and MtoA may not be the only mechanisms for neutrophilic iron oxidation. There are a number of additional uncharacterized cytochromes and electron transport genes (27, 42) within Gallionellaceae genomes such as isolate *S. lithotrophicus* ES-1 (27, 42), suggesting the existence of novel iron oxidation genes and mechanisms within the family.

The Gallionellaceae also includes a recently identified genus, *Candidatus* Nitrotoga, which are chemolithotrophic nitrite-oxidizing bacteria (NOB). Like the iron-oxidizing Gallionellaceae, they are widespread in freshwater and engineered environments, including permafrost (45), coastal sediments (46), freshwater (47), freshwater sediments (48), and the activated sludge of wastewater treatment facilities (49, 50). There are only two isolates, *Ca*. Nitrotoga fabula (49) and *Ca*. Nitrotoga sp. AM1P (51), along with four genomes from enrichment cultures (48). *Ca*. Nitrotoga are adapted to niches with low nitrite and oxidize it using a distinct high-affinity Nxr nitrite oxidoreductase (45, 48, 52). Extensive iron uptake mechanisms have been detected in *Ca*. Nitrotoga genomes, indicating the importance of iron for growth, likely due to the FeS cluster of Nxr (48). However, neither the isolates nor enrichments are known to oxidize Fe(II). If *Ca*. Nitrotoga lack the capacity to oxidize iron, then we can investigate the iron-oxidizing mechanisms and adaptations of Gallionellaceae through a comparative genomic analysis of iron- vs nitrite-oxidizing members.

Toward this goal, we took advantage of the growing number of environmental metagenomes and collected 103 Gallionellaceae genomes and metagenome assembled genomes (MAGs) with >80% completeness and <7% contamination. We used those sequences to resolve the Gallionellaceae phylogeny and delineate groups of iron and nitrite oxidizers. We searched for known and novel iron oxidation genes, other energy and nutrient metabolisms, and genes found exclusively in FeOB that may represent adaptations for an iron-oxidizing lifestyle. This work increases our understanding of Gallionellaceae family phylogeny and the metabolic traits of its genera. It also highlights some of the key multiheme cytochromes (MHCs) in Gallionellaceae FeOB, which may facilitate extracellular electron uptake and the oxidation of different iron substrates.

## RESULTS

### Phylogeny

**Fig 1:**
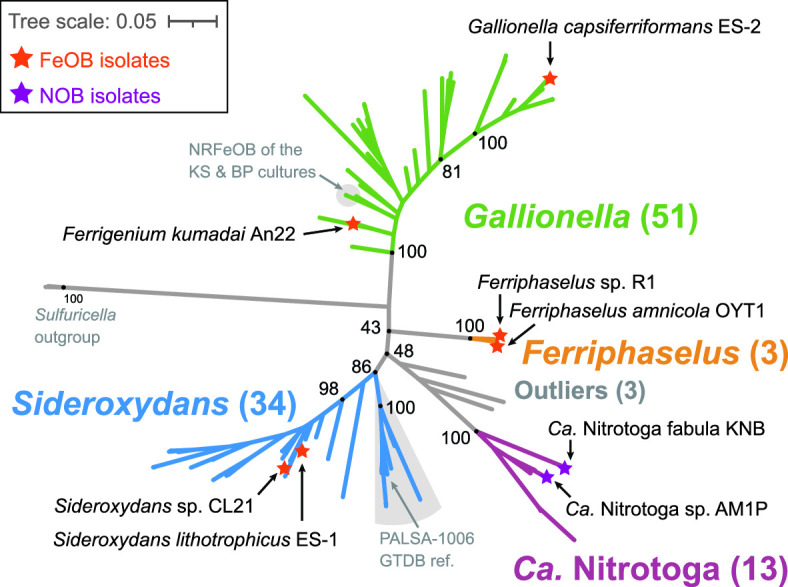
Concatenated ribosomal protein maximum likelihood tree of the Gallionellaceae family showing the four distinct genera: *Gallionella, Sideroxydans, Ferriphaselus*, and *Ca*. Nitrotoga. Isolates are labeled and annotated with stars. Numbers in parentheses indicate the number of genomes in each genus or group. Support values from 1,000 bootstraps are shown for major branching nodes (black dots). Detailed tree is shown in Fig. 2.

We collected 103 Gallionellaceae isolate genomes and MAGs with >80% completeness and <7% contamination from various databases and collections (Table S1). Many of these MAGs were only classified at the family level, so genus-level designations were initially unclear. To resolve the phylogeny, verify existing classifications, and classify the unknown Gallionellaceae, we examined 16S rRNA gene identity and constructed a concatenated protein tree (Fig. 1) from 13 ribosomal protein sequences. We also present genome average nucleotide identity (ANI) and amino acid identity (AAI) to further evaluate relatedness.

For genomes that have 16S rRNA sequences, we found the 16S percent identity between organisms ranges from 91.5% to 99.9% (Table S2a). Comparing the 16S percent identities of Gallionellaceae isolates (in bold in Table S2a) at a 94.5% or 95% identity threshold (53, 54), this suggests the existence of three genera (*Ferriphaselus*, *Gallionella-Ferrigenium*, and *Ca*. Nitrotoga-*Sideroxydans*). For whole genome comparisons, ANI and AAI range from 69.4% to 100% and 65.8% to 99.9%, respectively (Tables S2b and S2c), suggesting the whole family is closely related. These ANI and AAI values do not neatly cluster the genomes into distinct groups, though the values more clearly separate *Ca*. Nitrotoga and *Sideroxydans* than 16S (Table S2). However, there are no well-supported ANI or AAI thresholds or discontinuities that can delineate genera (55, 56).

In contrast, a concatenated protein tree (Fig. 1) using 13 ribosomal protein sequences shows distinct, well-supported clades that correspond to the four genera *Gallionella*, *Sideroxydans*, *Ferriphaselus*, and *Ca*. Nitrotoga (Fig. 1). Most of the MAGs previously classified as Gallionellaceae and Gallionellales were found to be either *Gallionella* or *Sideroxydans*, with the exception of one that clustered with the *Ca*. Nitrotoga (*Ca*. Nitrotoga SL_21). Although some genomes formed subclades, many were organized along a continuum. Near the base of the *Gallionella* are *Ferrigenium kumadai* An22 (25) and the nitrate-reducing FeOB (NRFeOB) of the Straub (KS) (30, 57) and Bremen Pond (BP) (32) enrichments (Fig. 1). There is not a clear boundary between the *Gallionella* and the relatively new *Ferrigenium* genus. In addition, the 16S percent identity between *F. kumadai* An22 and *Gallionella* species ranges from 94.7% to 95.5%, and the ANI and AAI values also indicate close relationships (Table S2). Therefore, we included the *Ferrigenium* and NRFeOB with the *Gallionella* grouping for our analyses. We also constructed a 16S rRNA gene tree containing 22 sequences in our data set along with 941 high-quality, full-length Gallionellaceae sequences from the SILVA database (Fig. S1), but bootstrap support was weaker, and clades were less clearly resolved. Therefore, concatenated ribosomal proteins are a more reliable determinant of Gallionellaceae phylogeny than 16S rRNA genes.

**Fig 2:**
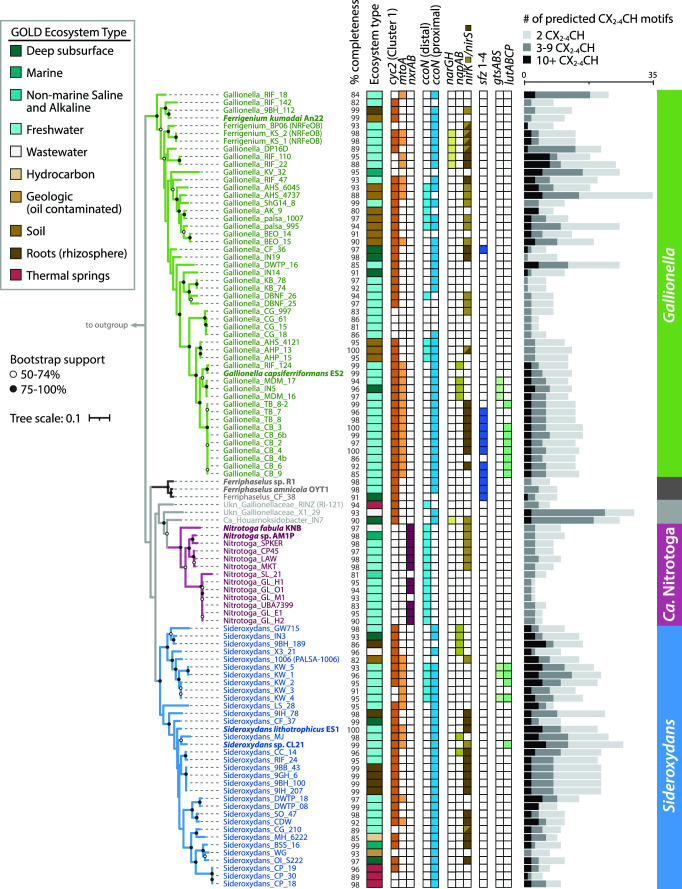
Maximum likelihood tree of concatenated ribosomal proteins from the Gallionellaceae, annotated with source ecosystem and genes for iron oxidation (*cyc2, mtoA*), nitrite oxidase (*nxrAB*), terminal oxidase (*ccoN*), denitrification (*narGH/napAB/nirK/nirS*), stalk formation (*sfz*), and organic utilization (*gtsABS*, *lutABCP*). The bar graph to the right shows the number of genes encoding multiheme cytochromes, categorized by number of CXXCH, CX_3_CH, and CX_4_CH heme-binding motifs. Phylogeny does not correlate to environments, and key genes, including those for multiheme cytochromes, show distinct distributions between iron and nitrite oxidizer clades. Isolates are shown in bold. % completeness = genome completeness calculated with CheckM. Outgroup omitted for space.

We assessed whether there was a relationship between phylogeny and environment. Each genome and MAG was classified with the Genomes OnLine Database (GOLD) classification schema (58) based on pre-existing GOLD classifications, available metadata, and publications (Fig. 2; Table S3). The majority of aquatic genomes were from freshwater and groundwater environments, while terrestrial genomes were mostly found in soil, peat, and rhizosphere environments. However, some genomes were sequenced from more extreme environments such as thermal hot springs (ENVO:00002181) and acid mine drainage (ENVO:00001997; Table S3). Gallionellaceae are widespread and can inhabit many different environments, but there was no clear pattern between GOLD Ecosystem Type and broad phylogenetic groupings (Fig. 2). Different Gallionellaceae appear to co-exist in some environments, suggesting niches not captured in the ecosystem classification are controlling Gallionellaceae diversity and environmental distribution.

### Metabolic potential and diversity

The Gallionellaceae family has few isolates, so to uncover the shared metabolic traits of its FeOB members, we compared and contrasted *Gallionella*, *Sideroxydans*, and *Ferriphaselus* genomes to those of the nitrite-oxidizing *Ca*. Nitrotoga. We identified key genes within the pangenome for iron oxidation (including predicted *c*-type cytochromes), carbon fixation, and respiration using a combination of Distilled and Refined Annotation of Metabolism (DRAM) (59), FeGenie (60), MagicLamp (61), a heme motif counter script (62), and BLAST (63, 64). To further uncover genes and pathways specifically enriched in the iron oxidizers, we used Anvi’o (65–67) to analyze a filtered data set of only *Gallionella*, *Sideroxydans*, and *Ca*. Nitrotoga genomes that were >97% complete. This approach enabled us to create a comprehensive picture of Gallionellaceae metabolic diversity and pinpoint promising gene clusters that may be adaptations for an iron-oxidizing lifestyle.

#### Primary energy metabolisms—iron and nitrite oxidation

**Fig 3:**
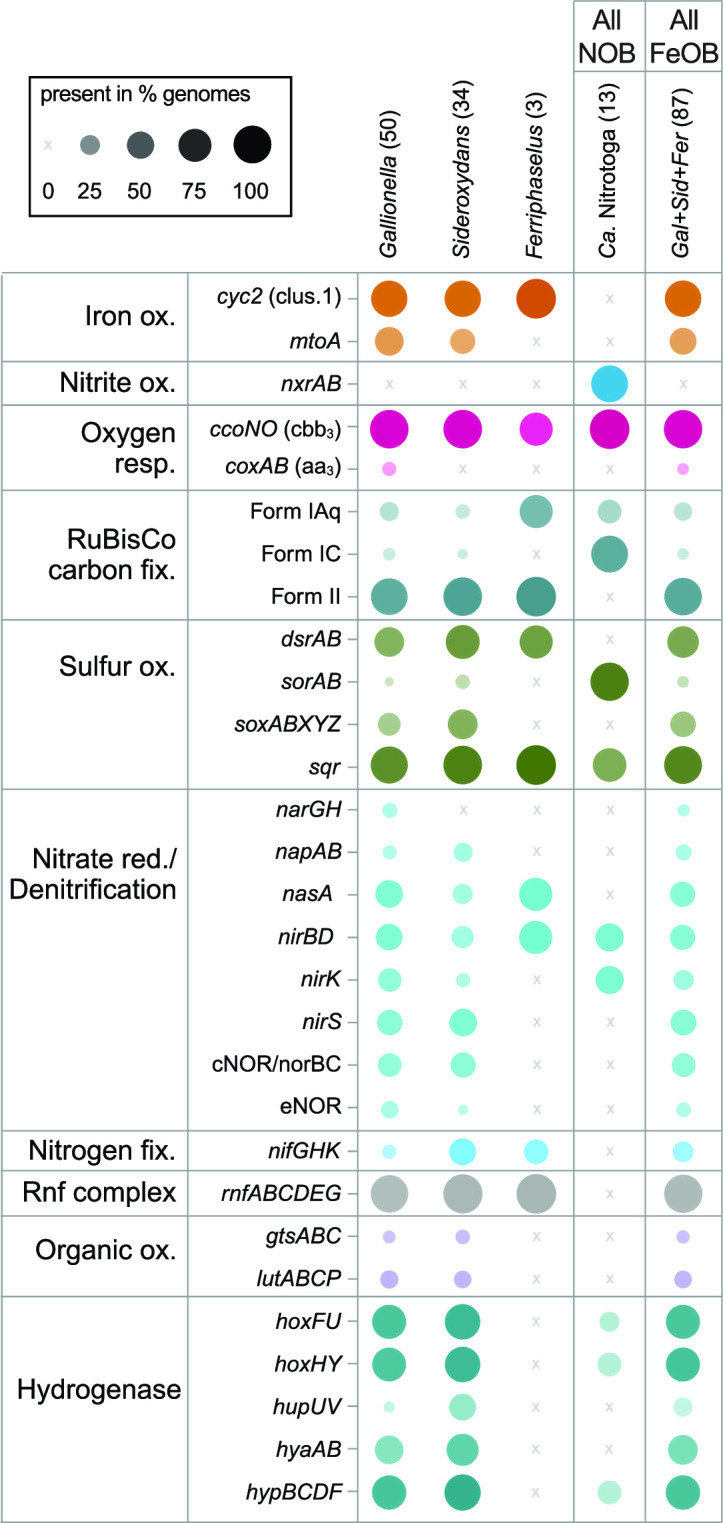
Plot showing the percent of genomes in each genus/group with genes for key metabolic pathways. The plot indicates the Gallionellaceae are aerobic lithoautotrophs with two main energy metabolisms, iron or nitrite oxidation. Some members also have metabolic potential for S oxidation and/or denitrification. Numbers in parentheses indicate the total number of genomes in each group. Color is used to distinguish groups, while dot size and opacity indicate % presence in the genome groups. Ox. = oxidation, red. = reduction, fix. = fixation, and resp. = respiration.

Known metabolisms for the few Gallionellaceae isolates suggest *Ca*. Nitrotoga are nitrite oxidizers, while *Sideroxydans*, *Ferriphaselus*, and *Gallionella* are iron oxidizers. We examined the pangenome for the presence of *cyc2* and *mtoA* iron oxidation genes and *nxrAB* nitrite oxidase genes to determine if that pattern also holds throughout the uncultured Gallionellaceae. As with the isolates, there is a clear delineation between organisms with marker genes for iron vs nitrite oxidation, which corresponds to the phylogenetic groups (Fig. 2 and 3).

The *cyc2* gene is widespread among clades of iron oxidizers, with at least one copy detected in 83% of the FeOB genomes (Fig. 3; Table S4). The *mtoA* gene is found in 41% of the FeOB genomes, and 37% of genomes have both *mtoA* and *cyc2*. In total, 89% have at least one iron oxidation gene, either *cyc2* or *mtoA* (Table S4). Since the data set includes multiple MAGs with a mean completeness score of 95%, it appears that almost all Gallionellaceae FeOB contain one of these two mechanisms for iron oxidation. Overall, *cyc2* homologs are more common than *mtoA* (Fig. 2 and 3), and some genomes encode multiple copies of *cyc2* (Table S4). All of the FeOB Gallionellaceae with *cyc2* encode at least one copy of a Cluster 1 Cyc2 [classified as in reference (35)]. Cluster 1 Cyc2 function has been verified through multiple lines of evidence [e.g., biochemistry (34), transcriptomics (27, 35), and proteomics (44)]. The Cyc2 sequences of the Gallionellaceae FeOB are closely related to each other and to a biochemically verified Cluster 1 Cyc2 iron oxidase (34). Therefore, we are confident in inferring that the Cluster 1 Cyc2 of Gallionellaceae is an iron oxidase.

The *Ca*. Nitrotoga SL_21 MAG contains only a predicted Cluster 2 Cyc2 homolog. Confidently assigning iron oxidation function to Cluster 2 Cyc2s depends on supporting context, which is lacking in this case. *Ca*. Nitrotoga SL_21 is not from a typical iron-oxidizing environment (permanently stratified, non-marine, saline lake), and although it is within Cluster 2, it is distant from the functionally verified Cyc2 representative from *Acidithiobacillus*. Currently, there is no clear evidence that this sole *Ca*. Nitrotoga Cyc2 is an iron oxidase.

In contrast, *nxrAB* genes are exclusive to the *Ca*. Nitrotoga. Copies of *nxrAB* are the most common genes for energy conservation among the *Ca*. Nitrotoga, present in 85% of the genomes (Fig. 2 and 3). Given that many of the genomes are MAGs with a mean completeness of 94%, the distribution of *nxrAB* appears to indicate that nitrite oxidation is the main energy metabolism of *Ca*. Nitrotoga. Thus, our pangenome analysis confirms Gallionellaceae can be divided into two main groups based on primary energy metabolism—FeOB and NOB.

#### *c*-type cytochromes

Both Cyc2 and MtoA are *c*-type cytochromes that transport electrons across the outer membrane. FeOB uses additional *c*-type cytochromes to transport electrons through the periplasm to the rest of the electron transport chain. We reasoned that novel iron oxidation mechanisms may also utilize *c*-type cytochromes, so we searched the Gallionellaceae genomes for proteins containing the CXXCH, CX_3_CH, and CX_4_CH heme-binding motifs (abbreviated hereafter as CXXCH). There is a stark difference between FeOB and NOB in the distribution of predicted *c*-type cytochromes. FeOB genomes have an average of 1.5× more CXXCH-containing proteins than NOB, and only the FeOB genomes encode proteins with 10 or more CXXCH motifs (Fig. 2). The abundance of genes for potential *c*-type cytochromes, in particular MHCs, suggests the presence of additional iron oxidation mechanisms in the Gallionellaceae FeOB.

**Fig 4:**
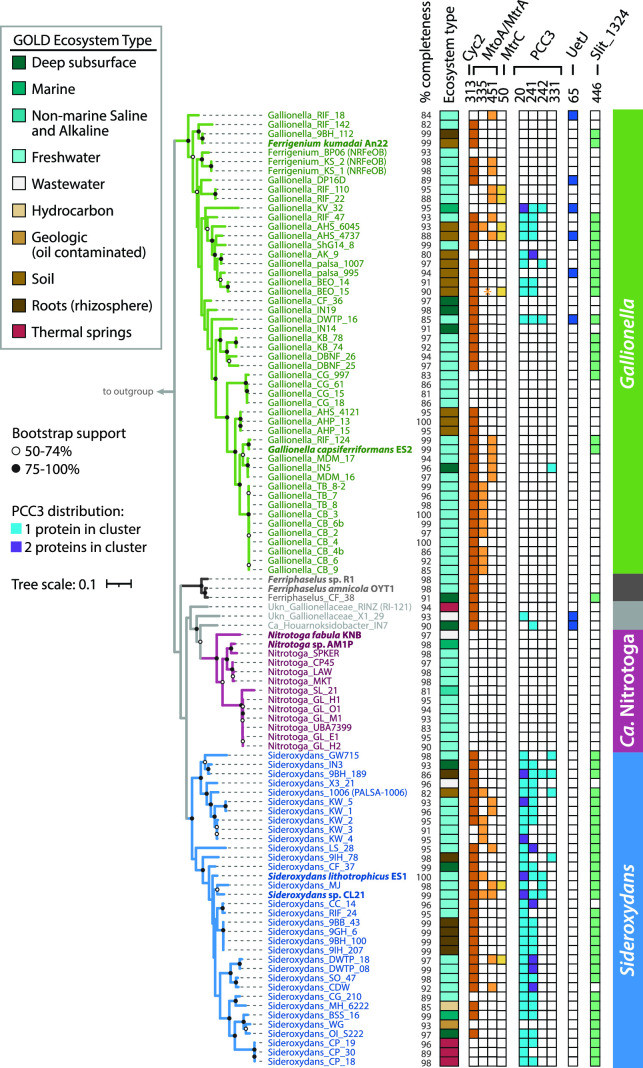
Maximum likelihood tree of concatenated ribosomal proteins from the Gallionellaceae that shows the distribution of MMSeqs2 clusters that represent predicted cytochromes Cyc2, MtoA, MtoC, PCC3, Uet, and Slit_1324. Asterisk (*) for Gallionella_BEO_15 indicates a partial MtoA sequence was detected using HMMs and verified with BLAST but was too short to bin into the MMseqs2 MtoA clusters. Isolates are shown in bold. % completeness = genome completeness calculated with CheckM. Outgroup omitted for space.

**Fig 5:**
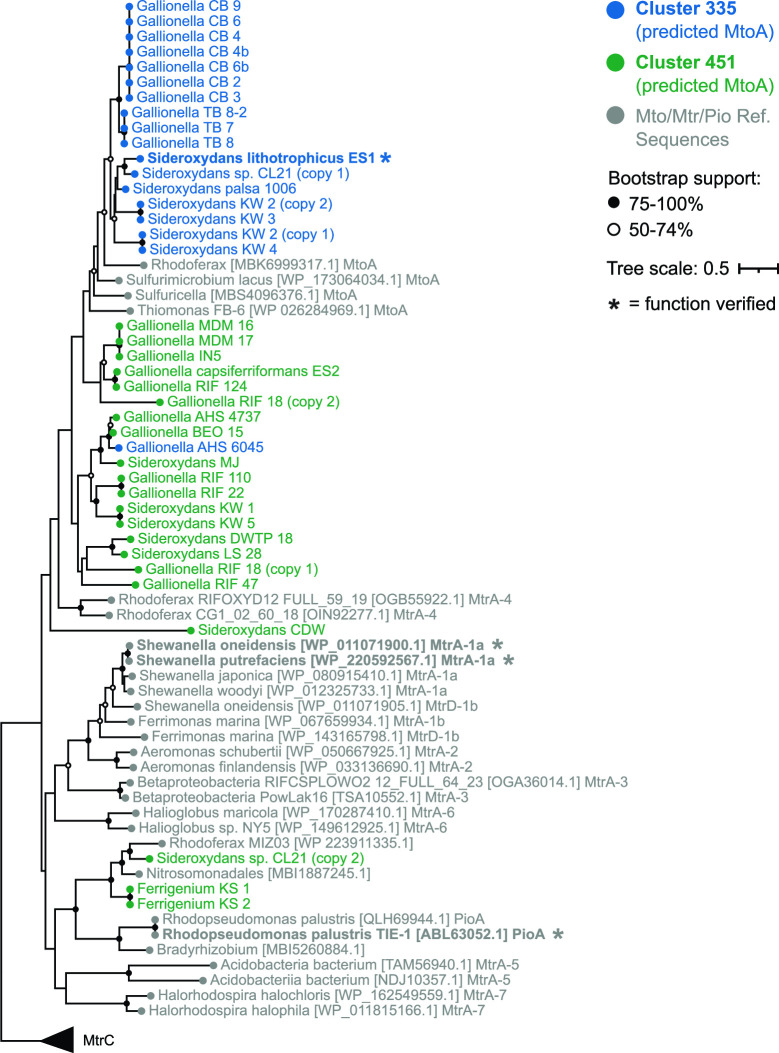
Maximum likelihood tree of the predicted MtoA sequences identified in MMSeqs2 Cluster 335 and Cluster 451 along with MtoA reference sequences from National Center for Biotechnology Information and MtrA reference sequences from Baker et al. 2022. Numbers (1a, 1b, 2, 3, 4, 5, 6, and 7) appended after Mtr denote reference sequences from the seven MtrA groups defined by Baker et al., 2022. MtrA-4 indicates the Group 4 Betaproteobacteria. Tree is rooted using MtrC. Support is the result of 500 bootstrap replicates.

To find *c*-type cytochromes of interest, all CXXCH-containing proteins were clustered using MMSeqs2 with bidirectional coverage and an 80% alignment cutoff. Clusters of sequences were then classified with representative sequences from isolates using BLAST to query the Uniprot database. If the cluster did not contain a sequence from an isolate, a consensus classification was used. A cluster of monoheme proteins (Cluster 313) was classified as Cyc2, and three clusters of decaheme proteins were classified as MtoA (Clusters 335 and 451) and MtrC (Cluster 50; Fig. 2 and 4; Table 1). These Cyc2, MtoA, and MtrC clusters largely agree with FeGenie’s HMM-based predicted distributions. Since MMSeqs2 generated two clusters of MtoA sequences, we sought to further verify the classifications. We constructed a tree of all Gallionellaceae MtoA sequences along with reference sequences of MtrA from FeRB (Fig. 5) (68). Although there is some separation of Cluster 335 and Cluster 451 MtoA sequences, many clades are not well defined or supported. In fact, backbone support throughout the tree is poor, and the tree does not indicate a clear separation of the MtoA and MtrA sequences (Fig. 5). There is some evidence that the direction of electron flow through Mto/Mtr can be reversible (33, 69, 70). So, it may be that the functions of MtoA and MtrA are interchangeable, and in fact, they may be indistinguishable proteins that can conduct electrons across the outer membrane in either direction.

**TABLE 1:** Clusters of predicted *c*-type cytochromes and other heme-containing proteins of interest from MMSeqs2^*a*^

Cluster	Functional prediction	# CXXCH, CX_3_CH, or CX_4_CH motifs per protein	# FeOB (of 87)
Iron oxidation/reduction proteins			
313	Iron oxidase Cyc2^‡^	1	70
451	Decaheme c-type cytochrome, DmsE family, MtoA^‡^	10	19
335	Decaheme c-type cytochrome, DmsE family, MtoA^‡^	10	17
50	Decaheme c-type cytochrome, OmcA/MtrC family^†^	10	7
Potential extracellular electron transport pathway proteins			
20	Cytochrome C family protein; potential periplasmic PCC3 subunit^‡^	21, 24, and 27	42
241	Cytochrome C family protein; potential extracellular PCC3 subunit^‡^	10, 11, 12, 13, 14, 15, 16, and 18	34
242	Cytochrome C family protein; potential extracellular PCC3 subunit^‡^	26, 28, 29, 33, and 35	7
331	Cytochrome C family protein; potential extracellular PCC3 subunit^‡^	15 and 17	5
65	Doubled CXXCH motif-containing protein; Cytochrome c3 family protein^†^, potential UetJ subunit	11 and 12	6
479	Tetraheme cytochrome—potential UetA subunit	4	6
330	Cytochrome C7 domain-containing protein; Triheme cytochrome—potential UetDEG subunit	3	5
94	Cytochrome C7 domain-containing protein; Triheme cytochrome—potential UetDEG subunit	3	5
446	Diheme cytochrome c^‡^—potential Slit_1324	2	51
Sensory proteins			
152	Methyl-accepting chemotaxis sensory transducer; YoaH ^‡^	1	54
40	Methyl-accepting chemotaxis sensory transducer with Pas/Pac sensor; Aerotaxis receptor ^‡^	1	43
400	Diguanylate cyclase with PAS/PAC sensor; Cyclic di-GMP phosphodiesterase Gmr ^‡^	1	36
Other			
433	2Fe-2S ferredoxin^‡^	1 and 2	72
360	4Fe-4S ferredoxin iron-sulfur binding domain protein	1	41
403	Forkhead-associated protein^‡^	10	27
146	Cytochrome c; Octaheme tetrathionate reductase^†^	8	25
253	Sulfite reductase, dissimilatory-type, subunit DsrJ^†^	3	17

^*a*^ Functional predictions are based on ‡ isolate annotations and NCBI BLAST or † BLAST of sequences from metagenomes in Uniprot.

The decaheme cytochrome MtrC is the extracellular partner of the iron-reducing MtrAB complex of *Shewanella* (71). The MtrAB complex is a homolog of the MtoAB complex of FeOB and can function in reverse to take up electrons (40). MtrC was thought to be exclusive to the Mtr complex of iron-reducers since MtrC homologs were not detected in FeOB isolates with MtoAB. However, we found seven MAGs within both *Gallionella* and *Sideroxydans* that encode MtrC (Table S4), leading us to question whether its function is restricted to iron reduction. Another possibility is that MtrC can also work in reverse and be part of an iron oxidation pathway. Differentiating these possibilities would require physiological testing of an FeOB isolate with MtrC, which does not currently exist.

**Fig 6:**
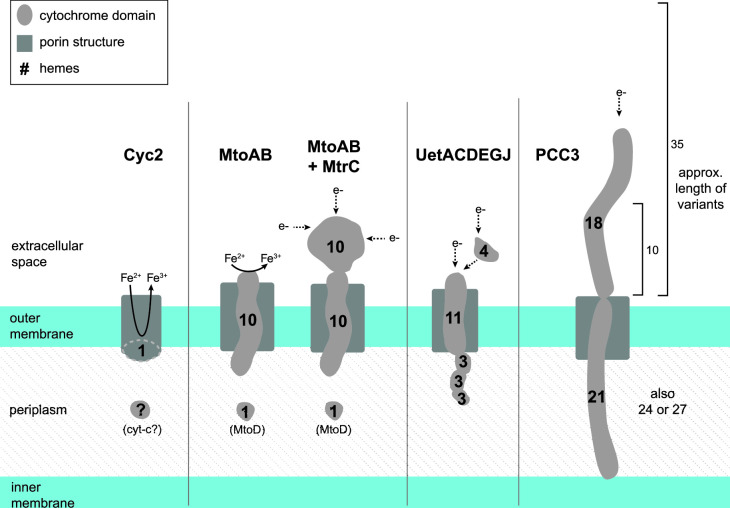
Models of potential Gallionellaceae extracellular electron transfer mechanisms. All sizes are approximated. Dimensions of Cyc2 with its fused cytochrome-porin and the porin-cytochrome complexes MtoAB, MtoAB+MtrC, MtoD, and Uet drawn from models and measurements in previous literature (34, 72–75). The illustration of PCC3 is based on AlphaFold2 predictions (Fig. **S2**). The number of hemes and size of PCC3 can vary. The 21/18 heme complex of *S. lithotrophicus* ES-1 is depicted along with the estimated length of the 10 and 35 heme variants of the extracellular cytochrome.

The Gallionellaceae FeOB have other porin-MHC complexes that could potentially catalyze iron oxidation, such as the PCC3 complex, identified through bioinformatic analyses of genomes of several FeOB including *S. lithotrophicus* ES-1. This predicted complex includes a periplasmic MHC, an extracellular MHC, an outer membrane porin, and a conserved inner membrane protein (72). We identified 26 Gallionellaceae FeOB genomes with a complete predicted PCC3 complex, an additional 11 genomes with a partial complex, and four instances where the PCC3 complex encodes two predicted periplasmic cytochromes instead of one (Fig. 4; Table S4). The predicted periplasmic MHCs grouped in MMSeqs2 Cluster 20, while predicted extracellular MHCs grouped in Clusters 241, 242, and 331. The extracellular MHCs exhibited variability in the number of CXXCH heme motifs (10–35; Table 1), which suggests a range of functions for the extracellular PCC3 MHCs. Based on *in silico* protein structure models, PCC3 MHCs appear long and mostly linear (Fig. 6; Fig. S2), suggesting an extended conduction range both intra- and extracellularly.

Another recently described porin-MHC complex is the undecaheme electron transfer (Uet) complex, found in the cathode-oxidizing Tenderiales (73) (Fig. 6). We used a combination of MMSeqs2 and BLAST to identify Uet genes in the Gallionellaceae. While PCC3 is more common to *Sideroxydans* (59%) than *Gallionella* (12%), the Uet pathway appears exclusive to *Gallionella* and two unclassified outliers (Fig. 4). Six *Gallionella* have predicted undecaheme cytochrome (UetJ), extracellular tetraheme cytochrome (UetA), three predicted periplasmic triheme cytochromes (UetDEG), peptidylprolyl isomerase (UetB), and NHL repeat units (UetHI; Fig. 4; Table S4). We checked for genes encoding the β-barrel porin UetC and found BLAST hits in four of the six genomes (Table S4).

*S. lithotrophicus* ES-1 has a set of periplasmic cytochrome genes without a predicted porin that was highly upregulated during growth on iron and, therefore, thought to be involved in iron oxidation (27). The genes encode a cytochrome b (Slit_1321), a hypothetical extracellular protein (Slit_1322), a monoheme cytochrome class I (Slit_1323), a periplasmic diheme cytochrome (Slit_1324; Cluster 446 in Table 1), and a molecular chaperone Hsp33 (Slit_1325). We found homologs of the Slit_1321–1324 genes are common, co-located, and well-conserved among Gallionellaceae FeOB, present in 50 genomes (Fig. 4; Table S4). These genes may represent a mechanism of periplasmic electron transport, perhaps as part of an iron oxidation/extracellular electron uptake pathway.

#### Electron transport chains

**Fig 7:**
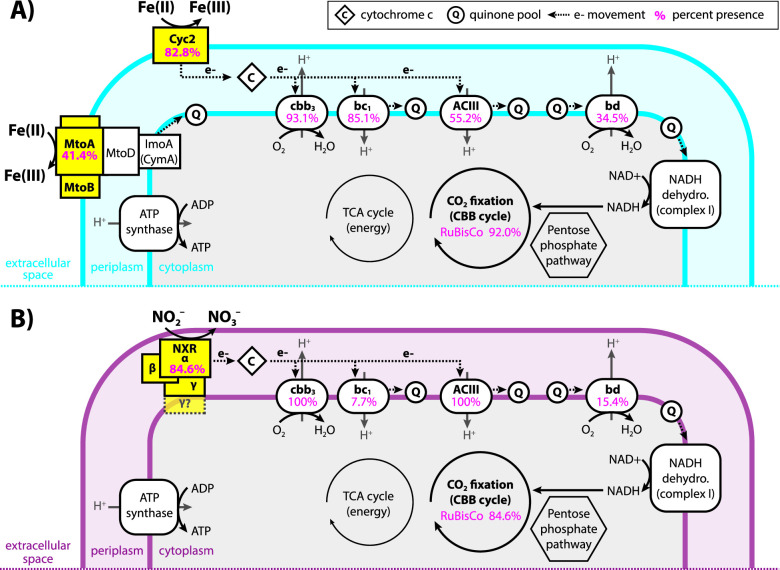
Diagram showing the similarities and differences between the electron transport chains of (**A**) iron- vs (**B**) nitrite-oxidizing Gallionellaceae. Pink numbers indicate the percent of FeOB (**A**) or NOB (**B**) genomes that encoded each part of the electron transport chain or RuBisCo. Less common components such as FeOB denitrification genes are not shown.

We compared electron transport chain component genes of the iron and nitrite oxidizer groups and found them to be largely similar (Fig. 7). High-affinity *cbb*_3_-type oxidases are common (Fig. 3), with most genomes containing either the proximal or distal form of *ccoN* (Fig. 2) (76). Even the four NRFeOB genomes contain *ccoNO* genes, indicating a potential for both oxygen and nitrate respiration. In contrast, few Gallionellaceae genomes contain *narGH* or *napAB* (6 and 10 genomes, respectively, with no overlap). Those that contain *narGH* include the known NRFeOB of the two separately maintained Straub cultures (*Ferrigenium straubiae* KS 1 and KS 2) (29, 57), three *Gallionella* MAGs, and one outlier (Table S4). This indicates the genetic potential for nitrate respiration is relatively rare overall (Fig. 3; Table S4).

In addition to the *cbb*_3_-type oxidase genes, 34.5% of iron oxidizers and 15.4% of nitrite oxidizers possess genes for cytochrome *bd*-type oxidases (*cydAB*) (Fig. 7). The presence of *bd*-type oxidase genes often overlaps with *cbb*_3_-type oxidase genes (Table S4). Like *cbb*_3_-type oxidases, cytochrome *bd*-type oxidases have a high affinity for oxygen, and recent studies show they can be more highly expressed than *cbb*_3_-type oxidases under low-oxygen, organic-rich conditions (77). Both FeOB and NOB have genes for cytochrome *bc*_1_ and Alternative complex III (ACIII) quinol oxidase complexes, which route electrons to the quinone pool where they may be used to form NADH for biosynthetic reactions. Genes for *bc*_1_ are more common in FeOB (85.1%) compared to NOB (7.7%), while ACIII is more common in NOB (100%) than FeOB (55.2%; Fig. 7; Table S4). Like the *bd*- and *cbb*_3_-type oxidases, the presence of *bc*_1_ and ACIII often overlaps in a single organism, especially in FeOB (Table S4). Possessing both *bd*- and *cbb*_3_-type oxidases and/or having both *bc*_1_ and ACIII contributes to flexibility within the electron transport chains of Gallionellaceae. The presence of various terminal oxidases implies adaptation to niches where oxygen and organic carbon availability differ or fluctuate.

#### Carbon fixation

Gallionellaceae isolates grow autotrophically. To determine if the capacity for autotrophic growth is widespread, we analyzed the pangenome for RuBisCo genes (*cbbLS*, *cbbMQ*). Most genomes in the data set (>91%; 94 of 103 genomes) contain genes for either Form I or Form II RuBisCo (Fig. 3; Table S4). FeOB more commonly have Form II, while NOB only have Form I. Form II enzymes are adapted for medium to high CO_2_ and low O_2_ concentrations (78), and their predominance in FeOB may correspond to different oxygen niches of FeOB and NOB. The prevalence of RuBisCo genes indicates both iron- and nitrite-oxidizing Gallionellaceae have the capacity to grow autotrophically.

#### Denitrification

Denitrification has been demonstrated in mixed cultures dominated by Gallionellaceae whose genomes encode various parts of the denitrification pathway (30–32). To better understand the denitrification potential across the Gallionellaceae and its role in FeOB metabolism, we analyzed patterns of denitrification genes (Fig. 2 and 3; Table S4). If autotrophic FeOB are to conserve energy from denitrification, they would in principle require the Nar nitrate reductase, as it is the only denitrification complex proven to generate proton motive force (79). Across our data set, genomes encoding NarGH are uncommon, found only in a cluster of five *Gallionella* (including the two *Ferrigenium* KS culture MAGs) plus the outlier *Ca*. Houarnoksidobacter (Table S4). These six genomes with *narGH* have at least one dissimilatory nitrite reductase (*nirK* or *nirS*), and four of the genomes encoded the eNOR nitric oxide reductase, giving the genetic capability to reduce nitrate to either NO or N_2_O, respectively. The eNOR nitric oxide reductase has a proposed proton channel, which may also contribute to energy conservation (80). Altogether, the results show a few genomes encode the ability to couple nitrate reduction to iron oxidation for energy, but this is rare amongst Gallionellaceae.

Gallionellaceae may denitrify for reasons other than energy generation, including N assimilation, redox balance, and removal of intermediates nitrite and nitric oxide, which are toxic and may abiotically oxidize Fe(II) (chemodenitrification). We found the denitrification genes *napAB*, *nasA*, *nirBD*, and *norBC* throughout the Gallionellaceae (Fig. 3; Table S4). The assimilatory nitrate reductase gene, *nasA*, was found exclusively in FeOB (Fig. 3; Table S3) co-located with nitrite reductase genes *nirBD*. A similar conserved gene cluster of *nasA* and *nirBD* has been observed in iron-oxidizing Zetaproteobacteria (81), allowing for the reduction of nitrate to ammonia for assimilation. Genomes with dissimilatory nitrate reductase genes *napAB* clustered in two small clades of *Gallionella* and *Sideroxydans* (Fig. 2; Table S4). NapAB may be used in aerobic denitrification when oxygen is limiting (82). The nitrite reductase genes *nirK/nirS* were more common than *napAB* and spread throughout the Gallionellaceae with little overlap (Fig. 2; Table S4). Both nitrite and NO present major challenges to FeOB metabolism because of their reactivity with iron: they bind to hemes, inhibiting the activity of cytochromes, and also directly oxidize Fe(II), thus competing with enzymatic iron oxidation (83). Therefore, it makes sense that many genomes have both *nir* genes and nitric oxide reductase (*nor*) genes (most commonly cNOR). Nitrous oxide reductase (*nosZ*) was not detected in any genomes, indicating Gallionellaceae are not able to fully denitrify to N_2_ gas; since N_2_O is non-toxic and relatively inert, *nosZ* is unnecessary.

Denitrification is a complex pathway that requires many enzymes and, therefore, a substantial investment of cellular resources. Given the high potential of Fe(II)/Fe(III) couples and the low-energy yield of iron oxidation, it may be difficult for autotrophic FeOB to compete with organoheterotrophic denitrifiers, which may explain the rarity of *nar* amongst strictly autotrophic FeOB. The ability to assimilate nitrate and detoxify intermediates is more common, suggesting the importance of these functions to FeOB.

#### Hydrogenases

Gallionellaceae genomes encode a variety of [NiFe]-hydrogenases (Fig. 3; Table S4). The most common type is the reversible, oxygen-tolerant hydrogenase, HoxFUHY, with genes present in 71% of FeOB and 30% of NOB. Other [NiFe]-hydrogenase genes were detected exclusively in FeOB, including genes for the oxygen-tolerant uptake hydrogenase, HyaAB, and the hydrogen-sensing hydrogenase, HupUV. [NiFe]-hydrogenases are capable of different physiological roles (84–87); thus, they may benefit in iron-oxidizing Gallionellaceae in multiple ways. Hydrogenases can enable the use of H_2_ as an electron donor, as in *Sideroxydans* sp. CL21 (28). Alternatively, the Hox hydrogenase can directly couple H_2_ oxidation to the reduction of NAD to NADH (84, 88) and generate reducing power for N_2_ fixation, CO_2_ fixation, and/or biosynthetic reactions. Hox can also function in reverse to transfer electrons from NADH to produce H_2_ as a mechanism of redox balance (89, 90), which can help FeOB cope with dynamic fluxes of Fe(II) and oxygen in redox transition zones.

#### Auxiliary energy metabolisms

Previous studies showed some Gallionellaceae FeOB possess alternate energy metabolisms such as thiosulfate and lactate oxidation (27, 28). We searched the pangenome for key genes of sulfur, manganese, and organic substrate oxidation pathways to determine how common alternate metabolisms are among Gallionellaceae FeOB. Sulfide:quinone reductase (*sqr*) is common to both FeOB and NOB (Fig. 3; Table S4). Sqr can oxidize sulfide, transporting electrons to the quinone pool, although it may be a means of detoxification rather than energy conservation (91, 92). In contrast, both *soxABXYZ* and *dsrAB* are detected exclusively in the iron-oxidizing Gallionellaceae genomes (Fig. 3; Table S4). To predict the oxidative vs reductive function of *dsrAB*, we constructed a tree using reference sequences from Loy et al. (93, 94). Gallionellaceae sequences form a discrete clade within the sulfur-oxidizing group (Fig. S3), indicating the DsrAB of Gallionellaceae is likely a reverse dissimilatory sulfite reductase. In contrast, the *Ca*. Nitrotoga genomes do not contain *dsr* or *sox* genes. Instead, *Ca*. Nitrotoga have *sorAB*, which may enable oxidation of sulfite to sulfate (Fig. 3). Gallionellaceae are not typically abundant in sulfur-rich environments. These results indicate sulfur oxidation is an auxiliary metabolism in Gallionellaceae with only certain FeOB capable of oxidizing S(0) or thiosulfate.

We analyzed the pangenome for signs of organic carbon utilization. Although not widely distributed, the most common genes were for lactate utilization (*lutABCP*) and sugar transport (*msmX, gtsABC*). Only eight *Gallionella* and five *Sideroxydans* genomes, including *Sideroxydans* sp. CL21, have *lutABC* along with the *lutP* lactate permease gene (Fig. 2 and 3; Table S4). Likewise, only six genomes contain *gtsABC* genes for glucose/mannose uptake. None of the NOB contain the *lut* or *gts* genes for organic carbon utilization.

We used BLAST to evaluate the Gallionellaceae genomes for manganese oxidase genes *mcoA*, *moxA*, *mofA*, and *mnxG*. There are a few hits for *mcoA*, *moxA*, and *mofA* genes but none for *mnxG* (Table S4). Since manganese oxidation activity has not been shown in any of the Gallionellaceae isolates, additional verification is needed to determine whether the genes identified by BLAST are truly Mn oxidases.

#### Other genes distinct to FeOB, potentially related to iron oxidation

We searched the pangenome for the candidate genes for stalk formation (*sfz/sfb*) identified in the stalk-forming *Ferriphaselus* and Zetaproteobacteria isolates (95, 96). The four *sfz/sfb* genes were found in 12 genomes, restricted to one crown-group cluster of nine *Gallionella* and all three *Ferriphaselus* (Fig. 2; Table S4). Thus far, all cultured Gallionellaceae stalk formers belong to these two genera, suggesting stalk formation may be limited and not a trait of *Sideroxydans*.

Using the Anvi’o subset of only genomes >97% complete, we identified several gene clusters that were present and abundant only in *Gallionella* and *Sideroxydans* but lacked a prior connection to an iron-oxidizing lifestyle. These included distinct gene clusters with Clusters of Orthologous Gene (COG) functional annotations for: Cell Wall/Membrane/Envelope Biogenesis, Cytoskeleton formation, Signal Transduction Mechanisms, and Energy Production and Conversion (see selected clusters in Table 2, and additional ones at https://doi.org/10.6084/m9.figshare.22781342). Clusters for Cell Wall/Membrane/Envelope Biogenesis may indicate FeOB have specific adaptations for housing extracellular electron transport mechanisms in the outer membrane or avoiding encrustation by iron oxides. Clusters for Energy Production and Conversion included ferredoxin (Fdx) and subunits of the RnfABCDEG complex. The Rnf complex was originally discovered for its role in N fixation, in which it oxidizes NADH and generates reduced ferredoxin that donates electrons to nitrogenase (97). More recent studies have shown Rnf complexes can conserve energy under anaerobic conditions (98–100), and as a low potential electron donor, ferredoxin can transfer electrons to many metabolic pathways including some that produce secondary metabolites (101). Not all Gallionellaceae with Rnf complex genes have *nifDHK* nitrogenase genes, implying Gallionellaceae Rnf and ferredoxin have functions beyond N fixation. Although their specific function in Gallionellaceae FeOB are unknown, their ubiquity implies utility for FeOB and an area for additional research.

**TABLE 2:** Gene clusters of interest from the Anvi’o pangenome subset that were present in iron-oxidizing *Gallionella* and *Sideroxydans* but absent in nitrite-oxidizing *Ca*. Nitrotoga (*Ferriphaselus* not considered)

COG category	COG function	Gene cluster ID
Cell wall/membrane/envelope biogenesis	Lipid carrier protein ElyC involved in cell wall biogenesis, DUF218 family (ElyC)	GC_00001120
	ABC-type lipoprotein export system, ATPase component (LolD)	GC_00000969
	ADP-heptose synthase, bifunctional sugar kinase/adenylyltransferase (RfaE)	GC_00001059, GC_00001084
	ADP-heptose:LPS heptosyltransferase (RfaF)	GC_00001100
	Glycosyltransferase involved in cell wall biosynthesis (RfaB)	GC_00001179
	Outer membrane protein TolC	GC_00000022, GC_00000920
	Glutamate racemase (MurI)	GC_00001047
	Murein L,D-transpeptidase YafK	GC_00001108
Cytoskeleton	Cytoskeletal protein CcmA, bactofilin family	GC_00000987
Energy production and conversion	Na^+^ translocating ferredoxin: NAD^+^ oxidoreductase RNF, RnfA	GC_00000042
	Na^+^ translocating ferredoxin: NAD^+^ oxidoreductase RNF, RnfB	GC_00001082
	Na^+^ translocating ferredoxin: NAD^+^ oxidoreductase RNF, RnfC	GC_00001069
	Na^+^ translocating ferredoxin: NAD^+^ oxidoreductase RNF, RnfD	GC_00001055
	Na^+^ translocating ferredoxin: NAD^+^ oxidoreductase RNF, RnfE	GC_00001071
	Na^+^ translocating ferredoxin: NAD^+^ oxidoreductase RNF, RnfG	GC_00001096
	Ferredoxin (Fdx)	GC_00001052
	Cytochrome *c*-type biogenesis protein CcmH/NrfF	GC_00001058
	Cytochrome *c*-type biogenesis protein CcmH/NrfG	GC_00001078
Signal transduction mechanisms	PAS domain | GAF domain | HAMP domain | Cyclic di-GMP metabolism protein	GC_00000006
	cAMP-binding domain of CRP or a regulatory subunit of cAMP-dependent protein kinases | Small-conductance mechanosensitive channel MscK	GC_00001152

## DISCUSSION

The Gallionellaceae family is historically known for its iron-oxidizing members, but recently, a new candidate genus of nitrite oxidizers, *Ca*. Nitrotoga, was identified (45). Comparing their genomes to those of FeOB genera has helped identify genes and pathways related to iron oxidation since *Ca*. Nitrotoga isolates have no documented capacity for that metabolism (45, 46, 48, 49, 51). We resolved the phylogeny of the Gallionellaceae and verified *Ca*. Nitrotoga lacked iron oxidation marker genes. Given separate groups of FeOB and NOB, we used a pangenomic approach to identify shared features of the Gallionellaceae, as well as FeOB-specific genes that may represent novel iron oxidation pathways.

### Phylogeny

Organizing and naming taxa is an essential step toward determining how microbial diversity is connected to function and niches, but it is a challenge to classify microbial taxa. The National Center for Biotechnology Information (NCBI) Taxonomy database and Genome Taxonomy Database (GTDB) place the Gallionellaceae into different higher-level taxa (Betaproteobacteria class and Nitrosomonadales order in NCBI, Gammaproteobacteria class and Burkholderiales order in GTDB). Each database also divides the family differently, with GTDB creating more genus-level classifications based on representative genomes for clades without cultured members. Here, we take a parsimonious approach to minimize the number of genera while maintaining nomenclature that has a long history of use in the literature. In this way, we can discuss groups with potentially distinct features and take advantage of previous findings without undue confusion. Because additional organisms are continuously discovered, we expect that this taxonomy will continue to evolve.

The Gallionellaceae can be described by four genera, *Gallionella, Sideroxydans*, *Ferriphaselus*, and *Ca*. Nitrotoga, based on the current concatenated ribosomal protein tree. Compared to this tree, 16S rRNA phylogeny did a poorer job of resolving these genera, so 16S-based identification should be considered tentative, pending the availability of genomes. ANI and AAI scores were not definitive, as there are no agreed-upon cut-offs to guide genus delineation (55, 56). Therefore, to facilitate consistent classification using the concatenated ribosomal protein phylogeny, the protein sequences and alignments used here (Fig. 1) are available at https://doi.org/10.6084/m9.figshare.21898938, https://doi.org/10.6084/m9.figshare.21898929.

The resolved phylogeny provides a framework for understanding the diversity and major metabolisms of the Gallionellaceae. They are members of Nitrosomonadales (or Burkholderiales), which contain many chemolithotrophic S and N oxidizers. Like their closest relatives, the Sulfuricellaceae (102), many Gallionellaceae retain the ability to oxidize sulfur (Fig. 3; Fig. S3). The Gallionellaceae tree (Fig. 1) shows a deeply branching split between genera, with each of the two major genera, *Gallionella* and *Sideroxydans*, containing a continuum of diversity. Within the *Gallionella*, the isolates *G. capsiferriformans* ES-2 and *Ferrigenium kumadai* An22 bracket the *Gallionella*, with An22 deeply branching and ES-2 at the crown. *F. kumadai* An22 was originally classified as *Ferrigenium* based on 16S rRNA distance (25). However, our analyses do not show any clear phylogenetic clustering or functional distinction, with which we could draw a line between *Gallionella* and *Ferrigenium*. Moreover, the tree topology suggests continued diversification within both *Gallionella* and *Sideroxydans* largely without the formation of subclades that represent distinct niches. There is one subclade of *Sideroxydans* that corresponds to the GTDB genus level designation PALSA-1006 (Fig. 1). However, 16S/ANI/AAI results (Table S2) indicate there is not enough diversity within the Gallionellaceae to justify further splitting the four major genera any further. Additionally, we did not detect any obvious functional difference in PALSA-1006. Given our phylogenetic analysis, 16S/ANI/AAI, and similar functional profiles, we recommend keeping them within *Sideroxydans*. Based on the above classification scheme, most of the genomes (84 of 103) fall into either *Gallionella* or *Sideroxydans*.

Phylogenetic diversity corresponds to functional diversity that can drive Gallionellaceae success in a variety of environments. Many *Gallionella* and *Sideroxydans* do not appear to be obligate iron oxidizers, and some may not be obligate aerobes. Auxiliary metabolisms for S, N, and C are present to varying degrees throughout the iron-oxidizing genera and are not associated with specific subgroups. Some FeOB from organic-rich environments, such as *Sideroxydans* sp. CL21, have genes for organoheterotrophy. Other FeOB show metabolic flexibility in additional lithotrophic metabolisms, such as oxidation of S or potentially Mn, elements that often co-occur with Fe in the environment. Some Gallionellaceae may also thrive in oxygen-poor environments by reducing nitrate, although this capability appears rare. Such traits contribute to diversity in the Gallionellaceae FeOB genera, which appear to acquire and/or retain additional energy and nutrient metabolisms to adapt to a range of environments.

*Ca*. Nitrotoga stands out as an exception within the Gallionellaceae. The pangenome analysis shows that *Ca*. Nitrotoga has distinctive genomic content (Fig. S4). They do not appear to have the capacity for iron oxidation based on available physiological evidence and the genomic analyses presented here. The similarities in Gallionellaceae FeOB and *Ca*. Nitrotoga electron transport chains enable them to meet the shared challenge of conserving energy from high-potential electron donors. However, *Ca*. Nitrotoga are a distinct clade that appears to have evolved from the FeOB to occupy a nitrite oxidation niche.

### Iron oxidation and extracellular electron uptake (EEU) mechanisms

The Gallionellaceae FeOB genomes encode a wide variety of predicted *c*-type cytochromes. Of these cytochromes, many appear to be associated with the outer membrane, implying a role in extracellular electron transport. Cyc2 is present in the majority of Gallionellaceae FeOB genomes, while MHCs Mto/Mtr, Uet, and PCC3 are less common, each with different distribution patterns (Fig. 4), suggesting the different cytochromes play distinct roles.

Cyc2 has been shown to oxidize dissolved Fe(II) (27, 34, 44, 103). The monoheme Cyc2 is a small fused cytochrome-porin, and since aqueous Fe^2+^ is common to many redox transition zones, it makes sense that most FeOB would retain and use the simplest tool. But in Earth’s various environments, iron is largely available as minerals (clays, oxides, and sulfides) and also bound to organics (e.g., humic substances). The decaheme MtoA has been shown to play roles in the oxidation of mineral-bound Fe(II), specifically Fe(II) smectite clay (44). As an MHC, MtoA may have multiple benefits that help in oxidizing minerals. MtoA has a large redox potential window [−350 to +30 mV (33, 37)], which could help with the oxidation of solids, like smectite (44), that also have a range of redox potentials [e.g., −600 to +0 mV for SWa-1 vs −400 to +400 mV for SWy-2 (104)], which change as mineral-bound iron is oxidized or reduced. Assuming the MtoA structure is similar to MtrA, the 10 hemes span the membrane, making a wire that conducts from extracellular substrates to periplasmic proteins (74, 105). The multiple hemes allow for the transfer of multiple electrons at a time (36). Some MAGs with *mtoAB* also encode the extracellular decaheme cytochrome MtrC. In *Shewanella*, the MtrCAB complex requires MtrC to reduce solid minerals (ferrihydrite), while MtrAB alone can only reduce dissolved Fe(III) and electrodes (71, 106, 107). Likewise, Gallionellaceae MtrC may help increase interactions with different minerals. Some Gallionellaceae FeOB may retain genes for both Cyc2 and MtoAB (with or without MtrC) to oxidize different Fe(II) substrates in their environments.

Like MtrCAB, the predicted PCC3 complex includes both periplasmic and extracellular MHCs and a porin. A key difference is that the PCC3 cytochromes often have more hemes than MtoA/MtrA and MtrC. The greater number of hemes may serve to store electrons, as in a capacitor. They may also conduct across a greater distance; the PCC3 periplasmic MHC, with 21–27 hemes, is potentially long enough to span the entire periplasm [as noted by Edwards et al. (108)]. Intraprotein electron transfer between hemes is rapid (109–111); therefore, the periplasm-spanning MHC of PCC3 may allow for faster electron transfer compared to complexes containing smaller periplasmic cytochromes like the monoheme MtoD. The extracellular PCC3 MHC contains between 10 and 35 hemes, which could extend further from the outer membrane compared to MtrC. Not only would this extend the range of electron transfer but may also be faster than a “wire” of smaller cytochromes [e.g., *Geobacter* hexaheme OmcS (112)]. Increasing oxidation rates via larger MHCs would allow FeOB to oxidize substrates faster. Given that Fe(II) is subject to abiotic oxidation under certain conditions and other organisms may compete for EEU, such kinetic advantages would give FeOB a competitive edge.

### Conclusions

Gallionellaceae, specifically *Gallionella*, are best known for lithoautotrophically oxidizing iron to make mineral stalks that come together to form microbial mats at groundwater seeps (18, 113, 114). Although this may contribute to an impression that the niche is relatively restricted, 16S rRNA sequencing of cultures and environmental samples has revealed both the diversity of Gallionellaceae as well as its prevalence across practically any freshwater and some brackish environments where Fe(II) and O_2_ meet. The pangenome shows that Gallionellaceae possess metabolic flexibility to use non-iron substrates, notably sulfur, and the MHCs likely also confer further metabolic capabilities that may help them occupy a range of different iron- and mineral-rich niches. Gallionellaceae thrive in aquifers, soil, and wetlands, all of which have substantial mineral content. Thus, the widespread ecological success of Gallionellaceae may well correspond to genomes that encode a range of iron oxidation mechanisms as well as adaptations for varied environments.

It is becoming clear that there are multiple ways to oxidize iron, though we have varying levels of evidence for gene/protein function (37, 115, 116). Validating iron oxidation genes/proteins is painstaking work due to challenging cultures, low yield, few genetic systems, and the fact that iron interferes with many molecular extractions and assays. And yet, there are likely even more iron oxidation mechanisms, so we need to be strategic about choosing genes/proteins for deeper characterization. Our pangenome analysis gives a wider view of the distribution and frequency of potentially novel iron oxidation genes, which will help us to prioritize investigations. Furthermore, the varied outer membrane-associated cytochromes inspire us to investigate relationships between structure and function. Why are there so many different multiheme cytochromes? Is there substrate specificity, kinetic advantages, battery-like functions, or some utility we have yet to consider? Addressing these questions will help us understand how these proteins and pathways shape microbial transformations of varied Earth materials.

## MATERIALS AND METHODS

### Data collection and curation

Gallionellaceae genomes were collected from the NCBI Entrez database (117), the Joint Genome Institute Integrated Microbial Genomes (IMG) database (118), and the European Nucleotide Archive at EMBL-EBI database [*Sideroxydans* sp. CL21, *Ca*. Nitrotoga fabula KNB, and the “IN” MAGs (17, 49, 119)] (Table S5). We also received non-public genomes from the Ménez Lab at the Université de Paris [three genomes reconstructed by Aurélien Lecoeuvre from the Carbfix study in Hengill, Iceland (2); metagenomes available at Sequence Read Archive SRR3731039, SRR3731040, SRR4188484, and SRR4188643], and the Banfield Lab at the University of California, Berkeley [three genomes reconstructed by Alex Probst from Crystal Geyser in Utah, USA (120); (Table S5)]. This initial 230-genome data set included isolate genomes, MAGs, and single-cell amplified genomes that were taxonomically classified as members of the Gallionellales order; Gallionellaceae family; or the *Gallionella*, *Sideroxydans*, *Ferriphaselus*, *Ferrigenium*, or *Ca*. Nitrotoga genera in their respective databases. Duplicate genomes were identified and removed if they had identical accession numbers, or their ANI were 100%. CheckM v1.1.2 (121) was used to assess genome quality. Genomes with lower than 80% completeness and greater than 7% contamination were removed from the data set. The final filtered data set, referred to as “the Gallionellaceae” or “the data set,” contained 103 genomes (Table S1; 2, 7, 11, 17, 23–26, 32, 42, 47–49, 51, 57, 95, 119, 120, 122–145), including six of the Gallionellaceae FeOB isolates. The seventh isolate, *Sideroxyarcus emersonii* (26), was not published at the time of our main analysis, but a supplemental of its key metabolic genes and MHCs (https://doi.org/10.6084/m9.figshare.22781912) shows it has similar patterns to *Sideroxydans*.

#### Naming conventions

To assign simple, unique names to the metagenomes, codes were appended to genus-level names based on sample location and bin IDs (Tables S1, S5 and S7). Isolates retained their own unique names. Organisms that were taxonomically classified in their original databases at the family Gallionellaceae or order Gallionellales were, if possible, classified at lower taxonomic levels using a combination of AAI, 16S rRNA (if available), classification through the GTDB Toolkit (146), and placement in the concatenated ribosomal protein tree (Fig. 1 and 2).

#### Ecosystem classifications

To assess whether metabolic diversity correlated to ecosystem type, each genome was assigned to an ecosystem based on the GOLD (147) schema which leverages Environmental Ontology classifications (148). A genome’s pre-existing classification from IMG was used if available. Genomes without prior classification were categorized based on published descriptions of their sample sites and “habitat” information listed in their database of origin. Based on the GOLD classifications (Table S3), genomes were examined for patterns of correspondence between ecosystems and phylogenetic and/or metabolic diversity.

### 16S rRNA analyses

Twenty-two of the 103 Gallionellaceae genomes contained 16S rRNA gene sequences. All sequences >1,450 bp (35 total) were aligned in Geneious v.10.2.6 (149) using MUSCLE (150). The percent identity of the 16S rRNA sequences is shown in Table S2a.

### Calculation of average amino acid and nucleotide identities

AAI and ANI were computed to assess the similarity of genomes in the curated data set (Table S2). AAI was calculated using CompareM (151). ANI was calculated using ANIcalculator v.1.0 (152). Prior to using ANIcalculator, tRNA and rRNA sequences were removed to prevent overinflation of ANI estimates (55, 152). AAI and ANI results were spot checked using the Kostas Lab AAI/ANI Matrix Tool (http://enve-omics.ce.gatech.edu/g-matrix/index) (153) to assure patterns were consistent. Final AAI and ANI tables were formatted using Microsoft Excel.

### Tree construction

#### Concatenated ribosomal protein tree

A concatenated tree of ribosomal proteins (Fig. 1) was constructed to determine the phylogenetic relationships of genomes in the Gallionellaceae data set. Two *Sulfuricella* genomes, *Sulfuricella* sp. T08 and *Sulfuricella* 3300027815, were included as an outgroup to root the tree. The use of a *Sulfuricella* outgroup was based on previous literature (154, 155), which identified *Sulfuricella* and other members of the Sulfuricellaceae family as near neighbors of Gallionellaceae. The concatenated sequences were composed of 13 small and large ribosomal proteins (L19, L20, L28, L17, L9_C, S16, L21p, L27, L35p, S11, S20p, S6, and S9) present in 94 or more of the 105 genomes including the outgroup. Protein sequences were aligned in Geneious v.10.2.6 (149) using MUSCLE (150). Ends of the alignments were manually trimmed, and regions with over 70% gaps were masked, after which sequences were concatenated. The tree was constructed using RAxML-NG v1.0.3 (156) with the maximum likelihood method, LG + G model, and 1,000 bootstraps. The final tree was visualized and annotated with iTOL (157). The ribosomal protein sequences used to construct the tree are available on FigShare (https://doi.org/10.6084/m9.figshare.21898938, https://doi.org/10.6084/m9.figshare.21898929).

#### 16S rRNA gene tree

We constructed a 16S rRNA gene tree (Fig. S1) composed of sequences from our data set combined with a selection of sequences from the SILVA database to determine how well 16S rRNA resolves Gallionellaceae phylogeny compared to the concatenated ribosomal protein tree. Full-length (~1,500 bp) 16S rRNA genes were retrieved from 22 of the Gallionellaceae genomes using Anvio’s “anvi-get-sequences-for-hmm-hits” command for “Ribosomal_RNA_16S.” These genes were aligned in SINA (158) along with Gallionellaceae sequences from the Silva database (159) that had >1,475 bp and >85–90 sequence quality score. The outgroup is composed of *Thiobacillus*, *Ferritrophicum*, *Sulfuricella*, *Sulfuriferula*, and *Nitrosomonas* sequences acquired from the Silva database. The final alignment contained 965 non-redundant sequences, and the alignment length was 1,500 positions after trimming and masking all sequence gaps greater than 70%. A maximum likelihood tree was constructed using RAxML-NG v1.0.3 (156) with the GTR+G model and 300 bootstraps. Family- and genus-level classifications from the SILVA database were used to annotate the tree in Iroki (160).

#### Individual protein trees

Trees for DsrAB (Fig. S3) and Mto/Mtr (Fig. 5) were constructed from Gallionellaceae protein sequences along with reference sequences from NCBI, Loy et al. and Baker et al. (68, 93). Sequences were aligned with MUSCLE (150), ends were manually trimmed, and regions with over 70% sequence gaps were masked in Geneious v.10.2.6 (149). For the Dsr tree, DsrA and DsrB sequences were concatenated. Trees were constructed using RAxML-NG v1.0.3 (156) with the LG+G model. Branch support for Mto/Mtr tree is based on 500 bootstraps, and support for the DsrAB tree is based on 300 bootstraps. The final trees were visualized and annotated with Iroki (160).

### Pangenome analysis

#### Metabolic gene analysis

We used DRAM v0.0.2 (59) within KBase (161), LithoGenie within MagicLamp (61), and FeGenie (60) to identify key metabolic genes indicative of various oxidation, respiration, and carbon utilization pathways. NCBI BLAST+ (64) was used to identify additional genes for eNOR, cNOR, SorAB, Mn oxidases, LutABCP, and stalk formation. We then analyzed the presence/absence of the metabolic genes and looked for patterns across the concatenated protein tree, between genera, and between FeOB and NOB.

#### MHC analysis

To identify potential *c*-type cytochromes, we used a modified heme counter script (62) to search for CXXCH, CXXXCH, and CXXXXCH motifs within the protein sequences of each genome. The search identified 5,929 protein sequences with one or more CX_2-4_CH-motifs. To determine which protein sequences were shared between genomes, sequences were clustered using MMSeqs2 (162) with coverage mode 0 for bidirectional coverage of at least 80% of the query and target sequences. Several clusters of interest were identified based on either the number of CX_2-4_CH-motifs in each sequence or the relative abundance of FeOB sequences in the cluster. Querying with BLASTp (63) against the Uniprot (163) database was used to classify sequences from clusters of interest, thereby identifying clusters of predicted *c*-type cytochromes. Isolate sequences were used as representative sequences for cluster classification. If a cluster did not contain an isolate sequence, a consensus classification was used. The subcellular localization of proteins was predicted using a combination of PSORTb v3.0.3 (164) and LocTree3 (165).

Some MHCs were predicted to be part of Mto, PCC3, or Uet porin-cytochrome complexes. Therefore, we wanted to determine if the genes for these MHCs were colocalized in their respective genomes with genes for β-barrel porins, periplasmic proteins, and inner membrane proteins previously identified in the literature (72, 73). We searched for the associated genes using BLASTp and amino acid reference sequences from *S. lithotrophicus* ES-1 (MtoB, MtoD, and CymA), *Gallionella* AHS-4737 (MtoC), and *Ca*. Tenderia electrophaga (UetBCDEFGHI). The locus tags of BLASTp hits were then compared to the locus tags of the MHCs to evaluate synteny and colocalization. The same method was used to determine if diheme *c*-type cytochromes from MMseqs2 cluster 446 which includes Slit_1324 were colocalized with a cytochrome b (Slit_1321), hypothetical extracellular protein (Slit_1322), monoheme cytochrome class I (Slit_1323), and molecular chaperone Hsp33 (Slit_1325).

#### PCC3 modeling

To model predicted PCC3 proteins, we used ColabFold: AlphaFold2 using MMseqs2 (166). Setting included using MSA mode “MMseqs2 (UniRef+environmental),” pair mode “unpaired+paired,” protein structure prediction with “AlphaFold2-ptm,” and complex prediction with “AlphaFold-multimer-v2” (167, 168). The best scoring model was rendered in PyMol v2.5.4 (169).

#### Anvi’o subset analysis

We used the Anvi’o v7 (65, 67) to build a pangenome database of all *Gallionella* (16) , *Sideroxydans* (15), and *Ca*. Nitrotoga (6) genomes that were over 97% complete (Fig. S4) to analyze for additional genes important to FeOB lifestyles. *Ferriphaselus* had too few representatives to define a meaningful core genome and was therefore omitted. Genes were clustered within the Anvi’o pangenome using a min-bit parameter of 0.5 and an mcl inflation parameter of 2. The Anvi’o pangenome was used to compare gene clusters across the data set and to bin: (i) near-core (found in >85% of genomes), (ii) accessory (found in >1 but <85% of genomes), and (iii) strain specific (found in a single genome) sets of gene clusters. Gene annotations were assigned in Anvi’o using Prodigal (170), and functional annotations for Anvi’o gene clusters were assigned using the NCBI’s Database of COGs (171, 172). Data tables of the binned Anvi’o gene clusters were analyzed to identify gene clusters found in the near-core genomes of *Gallionella* and *Sideroxydans* but absent in *Ca*. Nitrotoga.

## SUPPLEMENTAL MATERIAL

Supplemental Figures msystems.00038-23-s0001.pdf Figures S1–S4.

Supplemental Tables msystems.00038-23-s0002.xlsx Tables S1–S6.

ASM does not own the copyrights to Supplemental Material that may be linked to, or accessed through, an article. The authors have granted ASM a non-exclusive, world-wide license to publish the Supplemental Material files. Please contact the corresponding author directly for reuse.
