# Supplementary material for: Gallionellaceae pangenomic analysis reveals insight into phylogeny, metabolic flexibility, and iron oxidation mechanisms
Source: mSystems. 2023 Oct 26;8(6):e00038-23. doi: 10.1128/msystems.00038-23 (PMC10734462; doi:10.1128/msystems.00038-23)
Supplement: Supplemental Figures — Figures S1–S4. [file msystems.00038-23-s0001.pdf]

Supplemental Materials

Gallionellaceae pangenomic analysis reveals insight into phylogeny, metabolic flexibility, and iron oxidation mechanisms

Rene L. Hoover[a,b], Jessica L. Keffer[b], Shawn W. Polson[d,e], Clara S. Chan[a,b,c]
a Microbiology Graduate Program, University of Delaware, Newark, Delaware, USA
b Department of Earth Sciences, University of Delaware, Newark, Delaware, USA
c School of Marine Science and Policy, University of Delaware, Newark, Delaware, USA
d Department of Computer and Information Sciences, University of Delaware, Newark, Delaware, USA
e Center for Bioinformatics and Computational Biology, University of Delaware, Newark, Delaware, USA

*Corresponding author: Clara S. Chan: cschan@udel.edu

Number of pages: 7
Number of figures: 4
Number of tables: 6

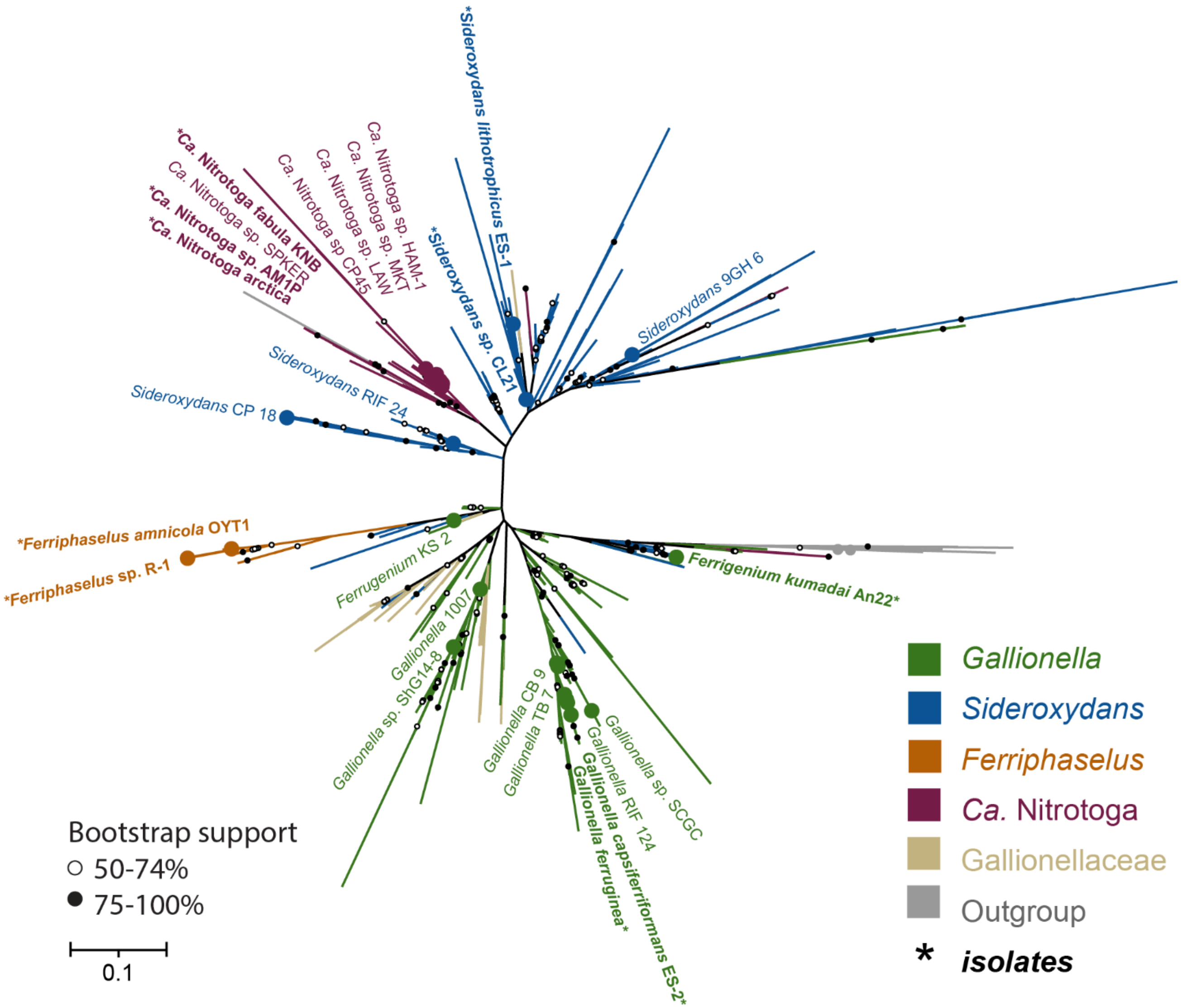

FIGURE S1 16S rRNA gene tree of the Gallionellaceae in this study along with 941 Gallionellaceae sequences from the Silva database (1) with >1475 bp and sequence quality scores >85%. Large colored circles indicate 16S rRNA sequences from genomes in the pangenome dataset. Asterisks next to bolded names indicate 16S rRNA from isolates.

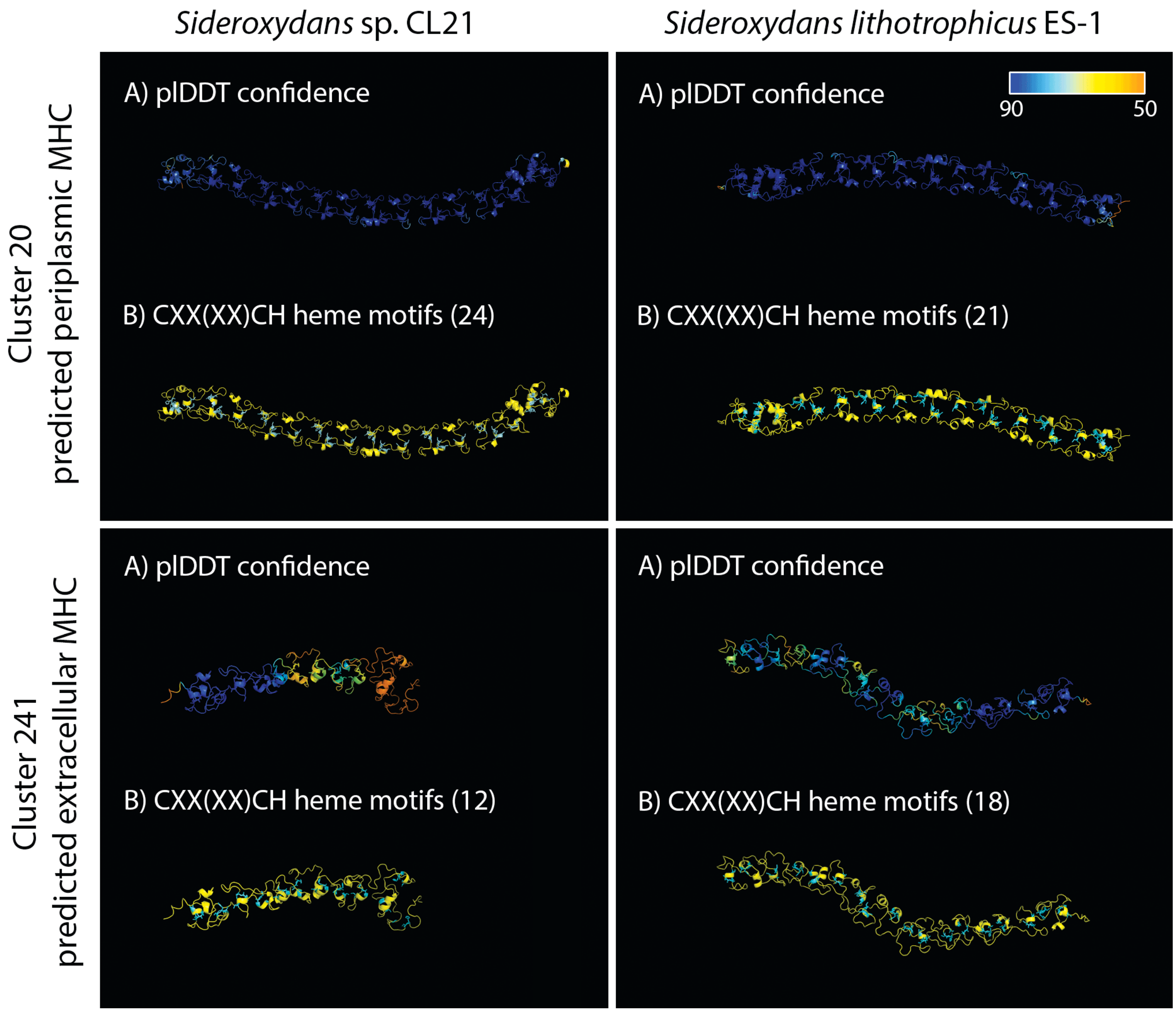

FIGURE S2 - AlphaFold2 (2, 3) models of predicted PCC3 proteins. Proteins colored A) by plDDT confidence with dark blue representing over >90% confidence and orange representing <50% confidence, and B) with the C and CH of predicted CXX(XX)CH motifs shown in cyan to represent the position of heme-binding within the yellow protein structure.

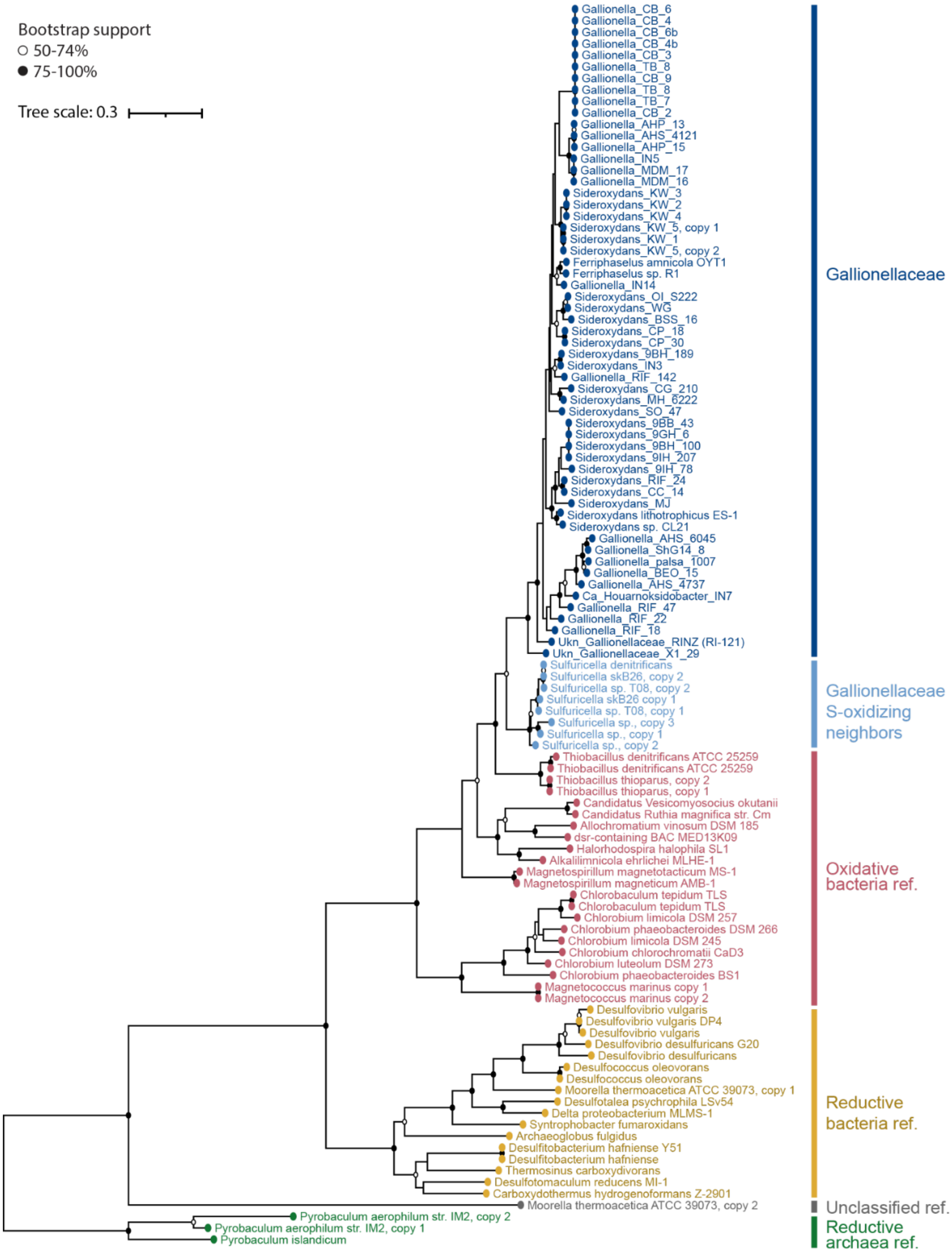

FIGURE S3 Concatenated DsrAB tree of Gallionellaceae sequences plus oxidative and reductive DsrAB reference sequences from Loy, et al. (4) and Müller, et al. (5). The tree shows Gallionellaceae and their S-oxidizing neighbors have oxidative rDSR.

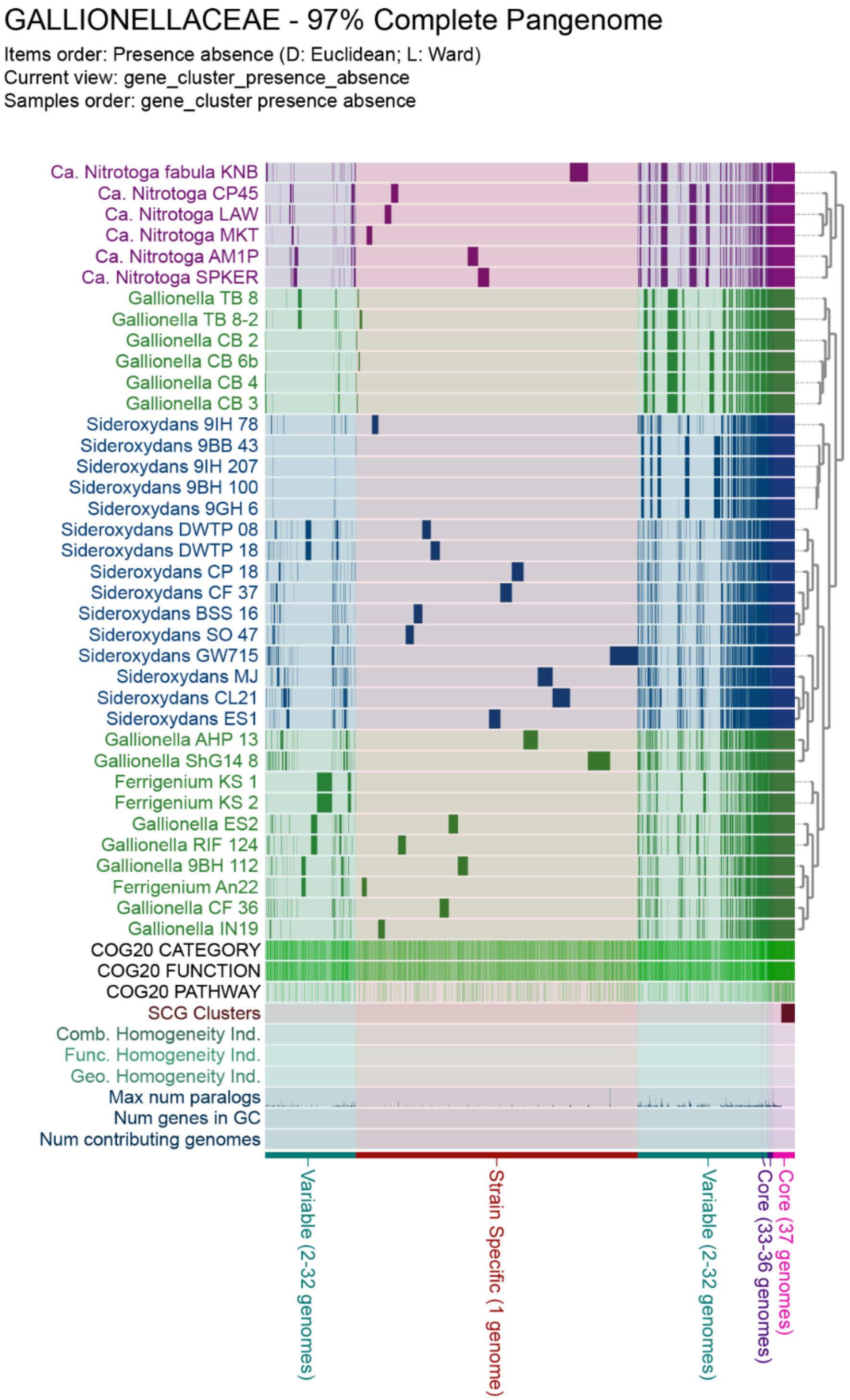

FIGURE S4 The visual representation of the Gallionellaceae pangenome in Anvi'o (6, 7) with all *Gallionella*, *Sideroxydans*, and *Ca.* Nitrotoga genomes over 97% complete. *Ferriphaselus* had too few representatives to define a meaningful core genome and is therefore omitted. Pangenome summary: # genomes = 37; # genes = 99,543; # gene clusters = 18,153; # gene clusters in all 37 genomes = 757 (29,404 gene calls in those clusters); # gene clusters in 33-36 genomes = 200 (7,428 gene calls in clusters); # gene clusters in 2-32 genomes (variable) = 7,531

(52,714 gene calls in those clusters); # gene clusters in 1 genome (strain specific) = 9,665 (9,997 gene calls in those clusters)

Supplemental Tables

TABLE S1 The table of chosen genomes with completeness, contamination, environmental and geographic metadata from databases and publications, and publication DOIs if applicable.
TABLE S2 16S % identity, ANI and AAI matrices for the Gallionellaceae dataset.
TABLE S3 Table of GOLD Ecosystem Classifications.
TABLE S4 Table of key metabolic genes.
TABLE S5 All considered genomes with databases and accession numbers.
TABLE S6 Naming conventions used for MAGs in this study.

References

1. Quast C, Pruesse E, Yilmaz P, Gerken J, Schweer T, Yarza P, Peplies J, Glöckner FO. 2013. The SILVA ribosomal RNA gene database project: improved data processing and web-based tools. Nucleic Acids Res 41:D590–D596.

2. Jumper J, Evans R, Pritzel A, Green T, Figurnov M, Ronneberger O, Tunyasuvunakool K, Bates R, Žídek A, Potapenko A, Bridgland A, Meyer C, Kohl SAA, Ballard AJ, Cowie A, Romera-Paredes B, Nikolov S, Jain R, Adler J, Back T, Petersen S, Reiman D, Clancy E, Zielinski M, Steinegger M, Pacholska M, Berghammer T, Bodenstein S, Silver D, Vinyals O, Senior AW, Kavukcuoglu K, Kohli P, Hassabis D. 2021. Highly accurate protein structure prediction with AlphaFold. 7873. Nature 596:583–589.

3. Evans R, O'Neill M, Pritzel A, Antropova N, Senior A, Green T, Žídek A, Bates R, Blackwell S, Yim J, Ronneberger O, Bodenstein S, Zielinski M, Bridgland A, Potapenko A, Cowie A, Tunyasuvunakool K, Jain R, Clancy E, Kohli P, Jumper J, Hassabis D. 2022. Protein complex prediction with AlphaFold-Multimer. bioRxiv https://doi.org/10.1101/2021.10.04.463034.

4. Loy A, Duller S, Baranyi C, Mußmann M, Ott J, Sharon I, Béjà O, Le Paslier D, Dahl C, Wagner M. 2009. Reverse dissimilatory sulfite reductase as phylogenetic marker for a subgroup of sulfur-oxidizing prokaryotes. Environmental Microbiology 11:289–299.

5. Müller AL, Kjeldsen KU, Rattei T, Pester M, Loy A. 2015. Phylogenetic and environmental diversity of DsrAB-type dissimilatory (bi)sulfite reductases. 5. ISME J 9:1152–1165.

6. Eren AM, Esen ÖC, Quince C, Vineis JH, Morrison HG, Sogin ML, Delmont TO. 2015. Anvi'o: an advanced analysis and visualization platform for 'omics data. PeerJ 3:e1319.

7. Eren AM, Kiefl E, Shaiber A, Veseli I, Miller SE, Schechter MS, Fink I, Pan JN, Yousef M, Fogarty EC, Trigodet F, Watson AR, Esen ÖC, Moore RM, Clayssen Q, Lee MD, Kivenson V, Graham ED, Merrill BD, Karkman A, Blankenberg D, Eppley JM, Sjödin A, Scott JJ, Vázquez-Campos X, McKay LJ, McDaniel EA, Stevens SLR, Anderson RE, Fuessel J, Fernandez-Guerra A, Maignien L, Delmont TO, Willis AD. 2021. Community-led, integrated, reproducible multi-omics with anvi'o. 1. Nature Microbiology 6:3–6.
